# Factors associated with influenza-like-illness: a crowdsourced cohort study from 2012/13 to 2017/18

**DOI:** 10.1186/s12889-019-7174-6

**Published:** 2019-07-04

**Authors:** Caroline Guerrisi, Marie Ecollan, Cécile Souty, Louise Rossignol, Clément Turbelin, Marion Debin, Thomas Goronflot, Pierre-Yves Boëlle, Thomas Hanslik, Vittoria Colizza, Thierry Blanchon

**Affiliations:** 1 Sorbonne Université, INSERM, Institut Pierre Louis d’Épidémiologie et de Santé Publique (IPLESP), F-75012 Paris, France; 2 Department of family Medicine, Faculté de Médecine, Université Paris Descartes, Paris, France; 3 APHP, Service de Médecine Interne, Hôpital Ambroise-Paré, 92100 Boulogne-Billancourt, France; 4 UFR des sciences de la santé Simone-Veil, Université de Versailles - Saint-Quentin-en-Yvelines, 78280 Versailles, France

**Keywords:** Human influenza, Epidemiology, Crowdsourcing, Cohort studies

## Abstract

**Background:**

Influenza generates a significant societal impact on morbidity, mortality, and associated costs. The study objective was to identify factors associated with influenza-like-illness (ILI) episodes during seasonal influenza epidemics among the general population.

**Methods:**

A prospective study was conducted with the GrippeNet.fr crowdsourced cohort between 2012/13 and 2017/18. After having completed a yearly profile survey detailing socio-demographic, lifestyle and health characteristics, participants reported weekly data on symptoms. Factors associated with at least one ILI episode per influenza epidemic, using the European Centre for Disease Prevention and Control case definition, were analyzed through a conditional logistic regression model.

**Results:**

From 2012/13 to 2017/18, 6992 individuals participated at least once, and 61% of them were women (*n* = 4258). From 11% (*n* = 469/4140 in 2013/14) to 29% (*n* = 866/2943 in 2012/13) of individuals experienced at least one ILI during an influenza epidemic. Factors associated with higher risk for ILI were: gender female (OR = 1.29, 95%CI [1.20; 1.40]), young age (< 5 years old: 3.12 [2.05; 4.68]); from 5 to 14 years old: 1.53 [1.17; 2.00]), respiratory allergies (1.27 [1.18; 1.37]), receiving a treatment for chronic disease (1.20 [1.09; 1.32]), being overweight (1.18 [1.08; 1.29]) or obese (1.28 [1.14; 1.44]), using public transport (1.17 [1.07; 1.29]) and having contact with pets (1.18 [1.09; 1.27]). Older age (≥ 75 years old: 0.70 [0.56; 0.87]) and being vaccinated against influenza (0.91 [0.84; 0.99]) were found to be protective factors for ILI.

**Conclusions:**

This ILI risk factors analysis confirms and further completes the list of factors observed through traditional surveillance systems. It indicates that crowdsourced cohorts are effective to study ILI determinants at the population level. These findings could be used to adapt influenza prevention messages at the population level to reduce the spread of the disease.

**Electronic supplementary material:**

The online version of this article (10.1186/s12889-019-7174-6) contains supplementary material, which is available to authorized users.

## Background

Seasonal influenza represents a major cause of morbidity and mortality worldwide, responsible for 3 to 5 million of serious illnesses [1], and for 290,000 to 650,000 deaths annually, according to recent updates from the World Health Organization and the United States Centers for Disease Control and Prevention [2]. Clinical manifestations occur through influenza-like-illness (ILI) with sudden onset of fever, myalgia and respiratory signs [3].

Documented influenza risk factors are related to *(i)* individual characteristics, such as age (higher risk of infection for young age, higher risk of complication and mortality for older age) [4, 5], immunodeficiency [1], pregnancy [6], chronic underlying medical conditions and respiratory diseases [7]; *(ii)* individual’s household features, such as living with children [8]; or *(iii)* individual’s profession like having contacts with children [9] or infected individuals [10]. However, the number of factors analyzed per study is often limited and mainly identified through traditional surveillance systems based on healthcare professionals [6, 7] or households follow-up [5, 8, 10].

Risk factors identified through healthcare systems or household studies may not be generalized to the general population, as they pertain more severe cases, or individuals selected based on their influenza susceptibility. Exploring risk factors for influenza directly from the general population can help to have a better knowledge of a larger spectrum of infections, including milder infections. Targeting message to individuals at risk of influenza and not only to those at risk of severe influenza may contribute to help limiting the spread of the disease in the population and potentially reducing the associated costs. An influenza epidemic in France is estimated to cost around $2.6 billion, with $0.3 billion of direct costs of medical care and $2.3 billion of indirect costs due to loss of productivity, leading to 2.9 (±2.5) days of work lost per influenza episode and person [11, 12].

Risk factors analyses based on the general population have been already addressed, but most were implemented during the 2009 A/H1N1pdm09 pandemic [13]. Since 2009, a participatory syndromic surveillance system for influenza, called Influenzanet, is operational in Europe [14, 15]. The system allows fine scale data collection among the general population enabling detailed risk factors analyses [16]. Here we focus on six consecutive influenza seasons, from 2012/13 to 2017/18, to estimate ILI frequency among the GrippeNet.fr (GN) cohort in France and identify the factors associated with ILI infections.

## Methods

### GrippeNet.fr data collection

GN is a crowdsourced surveillance system operating each year from November to April in mainland France since 2012. It is part of a broader European platform Influenzanet, where ten countries are involved [15]. Individuals from the GN cohort report their influenza-related symptoms through a dedicated website (https://www.grippenet.fr). Consent is informed and implied through registration and voluntarily completing a profile survey. This profile survey can be updated throughout the season, and covers socio-demographic (gender, age, household composition, occupation, place of residency); lifestyle (having pets, daily contacts, daily transportation means, smoking habit); and health-related characteristics (height and weight to estimate the body mass index (BMI), chronic treatments for at least one comorbidity including asthma, diabetes, immunosuppression, heart, kidney or pulmonary diseases, influenza vaccination status for the current and past seasons, respiratory allergies) [17, 18]. After the profile completion, symptoms data are collected on a weekly basis [14–16]. If symptoms are reported (from a list of 19 symptoms), further questions are asked to detail them and the participant behavior [19]. The profile and weekly symptoms surveys were published elsewhere (profile survey [17] and symptoms survey [19]). Participants can also answer to profile surveys and weekly symptoms surveys for other household members through multi-user accounts to facilitate for example participation and report of children and elderly. Each participant added in this way has all her/his individual information filled in the platform and was considered as an individual participant in our analyses.

Although GN participants were not representative of the French general population in terms of age and gender (overrepresentation of middle-aged individuals and women), all age classes were represented (data from 2011/12 season [17]). The GN population was also found to be more frequently employed, with a higher education level. No significant difference was found regarding chronic conditions, such as asthma and diabetes.

### Study period

The analysis was conducted on six GN seasons from 2012/13 to 2017/18.

### Study participants

Each season, GN participants who provided regular information were included in the current study. Regular information was defined as having filled at least one profile survey and three weekly symptoms surveys for a given season. At least one symptoms survey should have been completed before, another during and one after the influenza epidemic period as stated by the French national surveillance network in primary care, called *Sentinelles* network [20]. A similar inclusion criterion was used in a previous work conducted at the European level [16]. Individuals included in the study may have participated in one to six GN seasons.

### ILI case definitions

Different case definitions have been built from symptoms declared on GN [19]. For the risk factors analyses, we considered the ILI definition of the European Centre for Disease Control and Prevention (ILI_ECDC_), which is often used by other Influenzanet studies [16, 21, 22]. ILI is defined as (i) the sudden onset of symptoms, AND (ii) at least one of the following four systemic symptoms (fever or feverishness, malaise, headache or myalgia), AND (iii) at least one of the three respiratory symptoms (cough, sore throat, shortness of breath) [23]. The analyses were also conducted on a much more specific definition, closer to the one used by the French general practitioners (GP) *Sentinelles* network, stated as GN_ILI-_ and defined by (i) sudden onset of symptoms, AND (ii) fever ≥38 °C or fever (when the body temperature level was not available) AND pain or headache, AND (iii) sore throat or cough or shortness of breath [19]. The latter was computed as a sensitivity analysis, in order to clarify the ILI risk factors found.

As demonstrated in previous research, ILI represents a good proxy for influenza estimates [3, 24]. As no influenza virological confirmation was available, we took only into account ILI occurring during the influenza epidemic period identified by the *Sentinelles* network.

### Statistical analyses

Characteristics of the study participants between 2012/13 to 2017/18 were described depending on the last profile survey completed over the study period. As GN is an observational cohort, most of the participants are returning participants season after season, and so one participant can be counted in the study from one to six times. A person-season was defined as an individual having participated in the study during one season. Determinants associated with having at least one ILI episode during a follow-up season were estimated through a conditional logistic regression model, using the generalized estimated equations for longitudinal correlated data, based on the person-seasons. Explanatory variables (season, socio-demographic, geographic, health-related and lifestyle characteristics) were tested in univariate analyses. All covariates with a *p-*value below 0.2 were tested in multivariate analyses. Covariates were selected through a backward stepwise selection. The final model included all covariates associated with having at least one ILI episode with a *p*-value below or equal to 0.05. Same analyses were conducted using the more specific definition (GN_ILI-_). All statistical analyses were performed using the R software (3.2.5 version) and the *geepack* package [25, 26].

## Results

### Participation

Participation description is available in Table 1. Overall, there were 6992 individuals who participated at least once over the study period from 2012/13 to 2017/18. The 6992 individuals represented 24,653 person-seasons over the 6 year follow-up. An individual participated on average three seasons from 2012/13 to 2017/18. The mean number of participants per season was 4109 (min = 2943 in 2012/13 and max = 4780 in 2015/16). Individuals filled a mean number of 19 weekly symptoms surveys per season over the 6 years follow-up, with seasons lasting from 19 to 23 weeks.

**Table 1:** Participation and influenza-like-illness (ILI) cases reported per season from 2012/13 to 2017/18, GrippeNet.fr

	2012/13	2013/14	2014/15	2015/16	2016/17	2017/18
Epidemic periods (starting week-ending week; number of epidemic weeks)^a^	2012w51-2013w11 (13 weeks)	2014w05-2014w09 (5 weeks)	2015w03-2015w11 (9 weeks)	2016w04-2016w14 (11 weeks)	2016w50-2017w05 (8 weeks)	2017w50-2018w10 (13 weeks)
GN registered individuals^b^, N	6046	5907	6632	6515	6234	5958
GN participants included in the study^c^, n (%)	2943 (49%)	4140 (70%)	4428 (67%)	4780 (73%)	4204 (67%)	4158 (70%)
Symptoms surveys filled in by the included participants, N	58,235	76,578	80,106	99,333	73,711	82,252
Included participants having at least one ILI episode during the epidemic period (ILI_ECDC_ definition), n (%)	866 (29%)	469 (11%)	886 (20%)	829 (17%)	787 (19%)	855 (21%)
Included participants having at least one ILI episode during the epidemic period (GN_ILI-_ definition), n (%)	231 (8%)	111 (3%)	251 (6%)	203 (4%)	174 (4%)	198 (5%)

*GN* GrippeNet.fr

^a^Epidemic periods defined by the French *Sentinelles* network (available on http://www.sentiweb.fr/?page=epidemies)

^b^Any GN participants having filled in at least one profile survey

^c^Any GN participants having filled in at least three symptoms surveys (one before, one during and one after the influenza epidemic period)

### Description of the participants

Socio-demographic, lifestyle and health-related characteristics of the 6992 participants included in the study were described in Table 2. Overall, participants were mostly women (61%, *n* = 4258), living in urban area (81%, *n* = 5637), and having a mean age of 51 years old. Regarding daily contacts, 32% (*n* = 2268) were in contact with groups of ≥10 individuals, 26% (*n* = 1788) with children (beyond their own ones), 10% (*n* = 728) with patients and 10% (*n* = 721) with elderly. Vaccination against influenza for the current season was done in 36% (*n* = 2517) of the individuals followed (min = 29% in 2013/14, and max = 42% in 2017/18). Concerning the underlying health conditions, 22% (*n* = 1504) of the participants were treated for at least one comorbidity (as known as asthma, diabetes, immunosuppression, heart, kidney and pulmonary diseases). One third (*n* = 2413) had respiratory allergies and 11% (*n* = 766) were smokers. Further details are available in Table 2. The description of the participants for the six seasons from 2012/13 to 2017/18 did not show any difference between seasons. Full results are detailed in Additional file 1.

**Table 2:** Socio-demographic, exposure and health characteristics of GrippeNet.fr participants over the 2012/13 to 2017/18 study period and demographic characteristics of the French metropolitan population (Insee^a^, 2015 data). (For comprehensive results of the representativeness of GrippeNet.fr participants, see ref. [17])

	2012/13–2017/18 study period^b^ *N* = 6992	Metropolitan France general population (Insee^a^, 2015 data) *N* = 64,604,773
Sociodemographic characteristics		
Gender (m.d. = 0)		
Female	4258 (61%)	52%
Male	2734 (39%)	48%
Age (m.d. = 10)		
[0–5)	109 (2%)	6%
[5–15)	384 (5%)	12%
[15–45)	1900 (27%)	37%
[45–65)	2577 (37%)	26%
[65–75)	1567 (22%)	10%
75	445 (6%)	9%
Household composition (m.d. = 16)		
Living alone	1063 (15%)	36%
Living with ≥1 child	2435 (35%)	32%
Living with adults only	3478 (50%)	32%
Occupation (m.d. = 123)		
Working	3345 (49%)	40%
Student	774 (11%)	24%
Unemployed	160 (2%)	10%
Retired	2321 (34%)	25%
Stay at home/Sick leave	266 (4%)	–
Place of residency (m.d. = 0)		
Urban	5637 (81%)	77%
Rural	1355 (19%)	23%
Lifestyle		
Use of public transport (m.d. = 0)	1169 (17%)	–
Individuals’ contacts (m.d. = 0)		
Contact with patients	728 (10%)	–
Contact with elderly	721 (10%)	–
Contact with a group of people (≥10)	2268 (32%)	–
Contact with children	1788 (26%)	–
At least one pet at home (m.d. = 14)	3199 (46%)	–
Health characteristics		
Influenza vaccination (current season) (m.d. = 6)	2517 (36%)	–
Smokers (m.d. = 7)	766 (11%)	–
At least one health comorbidity^c^ (m.d. = 0)	1504 (22%)	–
Chronic treatment ^c^ for: (m.d. = 0)		
Asthma	441 (6%)	–
Diabetes	263 (4%)	–
Heart diseases	686 (10%)	–
Kidney diseases	50 (1%)	–
Immunosuppression	210 (3%)	–
Pulmonary diseases	195 (3%)	–
At least one respiratory allergy (m.d. = 0)	2413 (35%)	–
BMI (m.d. = 139)		
Underweight	348 (5%)	–
Normal weight	3946 (58%)	–
Overweight	1799 (26%)	–
Obese	760 (11%)	–

^a^The National Institute of Statistics and Economic Studies (https://www.insee.fr/fr/accueil) collects, analyses and disseminates information on the French economy and society

^b^Participants included in the analysis may have participated for the six GrippeNet.fr seasons. An individual may have participated up to six times (one time for each followed year). Here we analyze the participants’ characteristics depending on the last survey completed over the study period

^c^Participants receiving a chronic treatment for at least one of the following diseases: asthma, diabetes, immunosuppression, heart, kidney, and pulmonary diseases

*m.d.* missing data. The number corresponds to the total of missing data for a given variable among the 6992 participants

### Estimations of ILI episodes

Depending on GN season, from 11% (469/4140 in 2013/14) to 29% (866/2943 in 2012/13) of individuals experienced at least one ILI_ECDC_ episode during the influenza epidemic. Using the more specific GN_ILI-_ definition, this range reduced from 3% (111/4140 in 2013/14) to 8% (231/2943 in 2012/13) (Table 1).

### Risk factor analyses: univariate and multivariate analyses

In the univariate analysis, factors associated with having at least one ILI_ECDC_ episode during the influenza epidemic period with a *p*-value below 0.2 were: gender, age, household composition, occupation, use of public transport, pets at home, contacts with patients, contact with a group of individuals, contact with children, influenza vaccination status regarding the current and past seasons, being treated for health comorbidities, respiratory allergy, and body mass index (Table 3).

**Table 3:** Factors associated with at least one ILI episode (ILI_ECDC_) during six influenza epidemic periods, GrippeNet.fr

		N^a^	Cases^b^ n (%)	OR [CI 95%] Univariate analyses	*p*-value	OR [CI 95%] Multivariate analysis	*p*-value
Season	2012/13	2943	866 (29%)	*Ref.*	<10^−4^	*Ref.*	<10^− 4^
	2013/14	4140	469 (11%)	0.31 [0.27;0.34]		0.30 [0.26;0.33]	
	2014/15	4428	886 (20%)	0.59 [0.54;0.66]		0.60 [0.54;0.67]	
	2015/16	4780	829 (17%)	0.50 [0.45;0.55]		0.50 [0.45;0.56]	
	2016/17	4204	787 (19%)	0.54 [0.49;0.60]		0.57 [0.51;0.63]	
	2017/18	4158	855 (21%)	0.61 [0.55;0.68]		0.64 [0.57;0.71]	
Sociodemographic characteristics							
Gender	Male	9742	1623 (17%)	*Ref.*	<10^−4^	*Ref.*	<10^−4^
	Female	14,911	3069 (21%)	1.30 [1.20;1.40]		1.29 [1.20;1.40]	
Age	[45–65)	9940	1959 (20%)	*Ref.*	<10^−4^	*Ref.*	<10^− 4^
	[0–5)	306	111 (36%)	2.27 [1.78;2.89]		3.12 [2.05;4.68]	
	[5–15)	1133	279 (25%)	1.32 [1.12;1.55]		1.53 [1.17;2.00]	
	[15–45)	5948	1309 (22%)	1.15 [1.06;1.25]		1.13 [1.03;1.25]	
	[65–75)	6003	885 (15%)	0.71 [0.65;0.78]		0.88 [0.77;1.01]	
	≥75	1300	146 (11%)	0.54 [0.45;0.65]		0.70 [0.56;0.87]	
Household composition	Living alone	3917	731 (19%)	Ref.	<10^−4^	NS	NS
	Living with ≥1 child	7712	1714 (22%)	1.25 [1.12;1.40]			
	Living with adults only	12,953	2234 (17%)	0.92 [0.83;1.02]			
Occupation	Working	11,587	2432 (21%)	Ref.	<10^−4^	Ref.	<10^−2^
	Student	2162	543 (25%)	1.25 [1.11;1.41]		1.02 [0.83;1.26]	
	Unemployed	575	138 (24%)	1.18 [0.96;1.46]		1.18 [0.95;1.46]	
	Retired	8932	1299 (15%)	0.64 [0.59;0.70]		0.80 [0.70;0.91]	
						0.84 [0.70;1.02]	
	Stay at home/ Sick leave	993	191 (19%)	0.89 [0.74;1.07]			
Place of residency	Rural	4840	906 (19%)	*Ref.*	0.67	–	–
	Urban	19,813	3786 (19%)	1.02 [0.93;1.12]			
Lifestyle							
Use of public transport	No	20,843	3855 (18%)	*Ref.*	<10^−4^	*Ref.*	<10^−2^
	Yes	3810	837 (22%)	1.22 [1.12;1.34]		1.17 [1.07;1.29]	
Pets at home	None	13,492	2377 (17%)	*Ref.*	<10^−4^	*Ref.*	<10^− 4^
	At least one	11,118	2311 (20%)	1.21 [1.13;1.30]		1.18 [1.09;1.27]	
Contacts with patients	No	22,163	4154 (19%)	*Ref.*	0.01	NS	NS
	Yes	2490	538 (22%)	1.17 [1.04;1.30]			
Contacts with elderly	No	22,153	4234 (19%)	*Ref.*	0.41	–	–
	Yes	2496	458 (18%)	0.96 [0.86;1.07]			
Contacts with a group of people	No	16,795	3047 (18%)	*Ref.*	<10^−4^	NS	NS
	Yes	7858	1645 (21%)	1.17 [1.09;1.25]			
Contacts with children	No	18,722	3342 (18%)	*Ref.*	<10^−4^	NS	NS
	Yes	5931	1350 (23%)	1.33 [1.23;1.43]			
Health characteristics							
Influenza vaccination (current season)	No	16,083	3240 (20%)	*Ref.*	<10^−4^	*Ref.*	0.04
	Yes	8554	1450 (17%)	0.82 [0.76;0.88]		0.91 [0.84;0.99]	
Influenza vaccination (last season)	No	15,190	3083 (20%)	*Ref.*	<10^−4^	NS	NS
	Yes	9305	1575 (17%)	0.81 [0.75;0.87]			
Smoking status	Non smoker	21,995	4176 (19%)	*Ref.*	0.55	–	–
	Smoker	2630	514 (20%)	1.04 [0.92;1.16]			
Chronic treatment for at least one comorbidity^c^	No comorbidity	19,425	3648 (19%)	*Ref.*		*Ref.*	<10^−3^
	At least one comorbidity	5228	1044 (20%)	1.08 [0.99;1.17]	0.10	1.20 [1.09;1.32]	
	Asthma	1449	384 (27%)	1.53 [1.33;1.75]	< 10^−4^		
	Diabetes	941	156 (17%)	0.85 [0.70;1.04]	0.12		
	Heart diseases	2473	420 (17%)	0.88 [0.78;0.99]	0.03		
	Kidney diseases	152	24 (16%)	0.77 [0.48;1.25]	0.29		
	Immunosuppression	655	140 (21%)	1.13 [0.91;1.39]	0.26		
	Pulmonary diseases	635	161 (25%)	1.40 [1.13;1.73]	<10^−2^		
Respiratory allergy	None	16,400	2887 (18%)	*Ref.*	<10^−4^	*Ref.*	<10^−4^
	At least one	8253	1805 (22%)	1.29 [1.20;1.39]		1.27 [1.18;1.37]	
BMI	Normal weight [18.5;25[	14,000	2563 (18%)	*Ref.*	<10^−2^	*Ref.*	<10^−4^
	Underweight < 18.5	1053	201 (19%)	1.04 [0.88;1.25]		0.92 [0.77;1.11]	
	Overweight [25;30[	6571	1241 (19%)	1.04 [0.96;1.13]		1.18 [1.08;1.29]	
	Obese ≥30	2629	586 (22%)	1.26 [1.12;1.40]		1.28 [1.14;1.44]	

OR = Odd Ratio; CI95% = 95% confidence interval; NS = not significant; − = variable not tested in the multivariate analysis as the p-value was higher than 0.2 in univariate analysis

^a^*N* = global number of individuals for the six GrippeNet.fr seasons (from 2012/13 to 2017/18), based on the 24,653 person-seasons. An individual may have participated up to six times (one time for each followed year)

^b^The ILI case definition used here is the ILI_ECDC_ definition

^c^Participants receiving a chronic treatment for at least one of the following diseases: asthma, diabetes, immunosuppression, heart, kidney, and pulmonary diseases. Inclusion of the gathered variable “At least one comorbidity” only in the analyses.

In the final multivariate model (Table 3), women tended to have more ILI_ECDC_ episodes with respect to men (OR = 1.29, [1.20; 1.40]). Children experienced more ILI_ECDC_ episodes compared to adults from 45 to 64 (OR = 3.12 [2.05; 4.68] for children from 0 to 4, and OR = 1.53 [1.17; 2.00] for children from 5 to 14). Individuals using public transport (OR = 1.17 [1.07; 1.29]), having pets at home (OR = 1.18 [1.09; 1.27]), treated for health comorbidities (OR = 1.20 [1.09; 1.32]), having respiratory allergy (OR = 1.27 [1.18; 1.37]), or being overweight or obese (OR = 1.18 [1.08; 1.29] and OR = 1.28 [1.14; 1.44], respectively) were also more prone to experience an ILI_ECDC_ episode. On the contrary, individuals aged 75 years or older tended to have fewer ILI_ECDC_ episodes compared to those in the range of 45 to 64 (OR = 0.70 [0.56; 0.87]). Individuals vaccinated for the current season were also found to be at lower risk (OR = 0.91 [0.84; 0.99]).

The sensitivity analysis conducted with the more specific GN_ILI-_ definition showed similar results (see Additional file 2).

## Discussion

This study allowed us to identify factors associated with ILI directly from the general population. Some health determinants already described in the literature were found, such as having a young age or health comorbidities. We also found more debated risk factors, such as sex and the use of public transport, or rather unexpected ones, such as living with pets.

In our analyses, women were found to be at higher risk for ILI, independently of the case definition used (either ILI_ECDC_ or GN_ILI-_ definitions). Female vs. male differences regarding influenza have been previously evaluated: at younger (< 20 years) and older (> 80) ages, morbidity rates seem to be higher for males than females, however during the reproductive age (from 20 to 49 age group) women were found to have higher morbidity rates [27–29]. The observed differences are thought to be based on various factors that can affect both sex (e.g. genetic, immunological, and hormonal differences) and gender (i.e. behavioural) characteristics [27, 29, 30]. Here, the increased risk for women is found even adjusting for behavioural determinants such as living or having contacts with children. Further investigations should be implemented to understand better the biological and behavioural impacts.

Taking public transport is associated in our study with an increased risk for ILI. Only few studies addressed this association. One found that public transport used within 5 days of symptoms onset was associated with an increased risk of consulting for acute respiratory infection [31]. Previous Influenzanet works did not find an association between public transport and influenza [16, 21, 32], likely because of lack of statistical power due to the consideration of one season only [16, 21] or due to methodological differences based on the public transport covariate definition [32]. In this last article, the public transport covariate was defined using three categories (bicycle/foot, car and public transport), whereas here we opted for two categories (private vs. public transports) in order to better observe any impact of public transports with respect to other modes of daily locomotion, where individuals do not have close contacts.

Individuals who had pets at home had a higher chance of experiencing ILI as well. This result confirms previous findings obtained in the Influenzanet platform [16, 32], though it still remains unexplained. It would be interesting to further explore additional factors that can impact this small increased risk observed, such as the lifestyle of individuals living with pets and the contacts they establish.

In addition to the risk factor analysis, we estimated here the average fraction of individuals presenting at least one ILI episode during a season, ranging from 5% (GN_ILI-_) to 19% (ILI_ECDC_). International studies have provided estimates in the same ballpark (2.8 to 10.9% in the US [33], 10 to 25% in Canada [34]), however the comparison can be difficult as influenza impact depends on the specific season but also on the surveillance system and the case definition. In France, an average 3.4% of the population was estimated to have an ILI requiring a medical consultation from general practitioners surveillance data [20], a lower value compared to our GN estimate that can be explained by the limited fraction of illnesses leading to health-seeking behavior (56.7% of GN_ILI-_ episodes, 32.6% with the ILI_ECDC_ definition) [35].

The strength of this study is the identification of ILI determinants in the general population. Fine scale data have been collected through a large panel of individuals during several seasons allowing the evaluation of a wide range of ILI determinants. Little-known risk factors for ILI were identified in addition to well-known factors indicating that GrippeNet.fr is effective to study ILI determinants at the population level. However, a few limitations can be highlighted. First, the cohort was not representative of the French population as it is a crowdsourced system. Nevertheless, all ages and gender were represented. Second, no virological confirmation was available to ensure influenza follow-up. Thus, risk factors found here were not specifically associated with influenza infection but with a broader set of respiratory viruses causing similar symptoms. To limit the impact of this aspect, we decided to include only ILI episodes occurring during the influenza epidemic period, and also to explore two ILI case definitions, namely a more sensitive one (ILI_ECDC_) and a more specific one (GN_ILI-_).

## Conclusion

The identification of risk factors from the general population performed in this study confirms and further completes the list of factors observed through traditional surveillance systems. These findings can help target specific communication and influenza prevention campaigns at the population level aimed at reducing the spread of the disease. Some ILI risk factors, as gender, public transport use and having pets are still debated, they should be further investigated.

## Additional files

Additional file 1: Socio-demographic, exposure and health characteristics of GrippeNet.fr participants from seasons 2012/13 to 2017/18. This table represents the description of the participants for each of the six seasons studied. (DOCX 26 kb)

Additional file 2: Factors associated with at least one ILI episode (GNILI-) during six influenza epidemic periods, GrippeNet.fr. This table highlights the factors associated with an ILI episode using a more specific definition. (DOCX 25 kb)

## Data Availability

GrippeNet.fr databases used in this study are not publicly available, in accordance with the authorization we have from the French National Commission on Informatics and Liberty (CNIL, authorization DR-2012-024). The datasets used and/or analysed during the current study are available from the authors on reasonable request.

## Abbreviations

BMI: body mass index
ECDC: European Centre for Disease Prevention and Control
GN: GrippeNet.fr
ILI: influenza-like-illness
