# Supplementary material for: Factors associated with influenza-like-illness: a crowdsourced cohort study from 2012/13 to 2017/18
Source: BMC Public Health. 2019 Jul 4;19:879. doi: 10.1186/s12889-019-7174-6 (PMC6610908; doi:10.1186/s12889-019-7174-6)
Supplement: Supplementary file 1 — Socio-demographic, exposure and health characteristics of GrippeNet.fr participants from seasons 2012/13 to 2017/18. This table represents the description of the participants for each of the six seasons studied. (DOCX 26 kb) [file 12889_2019_7174_MOESM1_ESM.docx]

**Additional file 1.** Socio-demographic, exposure and health characteristics of GrippeNet.fr participants from seasons 2012/13 to 2017/18

|  | 2012/2013  N = 2,943 | 2013/2014  N = 4,140 | 2014/2015  N = 4,428 | 2015/2016  N = 4,780 | 2016/2017  N = 4,204 | 2017/2018  N = 4,158 |
| --- | --- | --- | --- | --- | --- | --- |
| Socio-demographic characteristics | | | | | |  |
| Gender (m.d.=0)  Female  Male | 1,838 (62%)  1,105 (38%) | 2,498 (60%)  1,642 (40%) | 2,671 (60%)  1,757 (40%) | 2,861 (60%)  1,919 (40%) | 2,539 (60%)  1,665 (40%) | 2,504 (60%)  1,654 (40%) |
| Age (m.d.=24)  [0-5)  [5-15)  [15-45)  [45-65)  [65-75)  ≥75 | m.d.=7  42 (1%)  96 (3%)  818 (28%)  1,270 (43%)  617 (21%)  93 (3%) | m.d.=3  58 (1%)  233 (6%)  1,134 (27%)  1,728 (42%)  840 (20%)  144 (3%) | m.d.=2  55 (1%)  221 (5%)  1,134 (26%)  1,793 (40%)  1,025 (23%)  198 (4%) | m.d.=7  59 (1%)  257 (5%)  1,154 (24%)  1,865 (39%)  1,184 (25%)  254 (5%) | m.d.=1  45 (1%)  174 (4%)  900 (21%)  1,664 (40%)  1,155 (27%)  265 (6%) | m.d.=3  47 (1%)  152 (4%)  808 (19%)  1,620 (39%)  1,182 (28%)  346 (8%) |
| Household composition (m.d.=71)  Living alone  Living with ≥1 child  Living with adults only | m.d.=6  448 (15%)  993 (34%)  1,496 (51%) | m.d.=12  593 (14%)  1,424 (34%)  2,111 (51%) | m.d.=14  685 (16%)  1,426 (32%)  2,303 (52%) | m.d.=18  751 (16%)  1,504 (32%)  2,507 (53%) | m.d.=11  707 (17%)  1,229 (29%)  2,257 (54%) | m.d.=10  733 (18%)  1,136 (27%)  2,279 (55%) |
| Occupation (m.d. = 404)  Working  Student  Unemployed  Retired  Stay at home/Sick leave | m.d.=28  1,429 (49%)  248 (9%)  57 (2%)  1,018 (35%)  163 (6%) | m.d.=64  2,018 (50%)  424 (10%)  100 (2%)  1,354 (33%)  180 (4%) | m.d.=82  2,098 (48%)  417 (10%)  122 (3%)  1,536 (35%)  173 (4%) | m.d.=90  2,238 (48%)  461 (10%)  119 (3%)  1,705 (36%)  167 (4%) | m.d.=75  1,937 (47%)  330 (8%)  101 (2%)  1,606 (39%)  155 (4%) | m.d.=65  1,867 (46%)  282 (7%)  76 (2%)  1,713 (42%)  155 (4%) |
| Place of residency (m.d. = 0)  Urban  Rural | 2,336 (79%)  607 (21%) | 3,312 (80%)  828 (20%) | 3,582 (81%)  846 (19%) | 3,863 (81%)  917 (19%) | 3,383 (80%)  821 (20%) | 3,337 (80%)  821 (20%) |
| Lifestyle |  |  |  |  |  |  |
| Use of public transport (m.d. = 0) | 438 (15%) | 665 (16%) | 682 (15%) | 750 (16%) | 648 (15%) | 627 (15%) |
| Individuals’ contacts (m.d. = 0)  Contact with patients  Contact with elderly  Contact with a group of people (≥10)  Contact with children | 297 (10%)  256 (9%)  943 (32%)  730 (25%) | 452 (11%)  419 (10%)  1,452 (35%)  1,097 (26%) | 442 (10%)  441 (10%)  1,407 (32%)  1,103 (25%) | 459 (10%)  478 (10%)  1,489 (31%)  1,155 (24%) | 430 (10%)  452 (11%)  1,315 (31%)  954 (23%) | 410 (10%)  450 (11%)  1,252 (30%)  892 (21%) |
| At least one pets at home (m.d. = 43) | m.d.=7  1,371 (47%) | m.d.=10  1,942 (47%) | m.d.=4  2,018 (46%) | m.d.=8  2,114 (44%) | m.d.=7  1,853 (44%) | m.d.=7  1,820 (44%) |
| Health characteristics |  |  |  |  |  |  |
| Influenza vaccination (current season) (m.d.=16) | m.d.=1  868 (30%) | m.d.=5  1,203 (29%) | m.d.=3  1,407 (32%) | m.d.=0  1,694 (35%) | m.d.=4  1,633 (39%) | m.d.=3  1,749 (42%) |
| Influenza vaccination (last season) (m.d.=158) | m.d.=23  978 (33%) | m.d.=28  1,371 (33%) | m.d.=25  1,594 (36%) | m.d.=23  1,803 (38%) | m.d.=25  1,721 (41%) | m.d.=34  1,838 (45%) |
| Smokers (m.d.=28) | m.d.=3  356 (12%) | m.d.=10  474 (11%) | m.d.=7  474 (11%) | m.d.=4  506 (11%) | m.d.=2  436 (10%) | m.d.=2  384 (9%) |
| At least one health comorbidity*  (m.d. = 0) | 563 (19%) | 811 (20%) | 893 (20%) | 1,048 (22%) | 915 (22%) | 998 (24%) |
| Chronic treatment * for: (m.d. = 0)  Asthma  Diabetes  Heart diseases  Kidney diseases  Immunosuppression  Pulmonary diseases | 176 (6%)  114 (4%)  254 (9%)  10 (0%)  67 (2%)  59 (2%) | 245 (6%)  145 (4%)  371 (9%)  25 (1%)  89 (2%)  95 (2%) | 249 (6%)  152 (3%)  430 (10%)  22 (0%)  120 (3%)  110 (2%) | 292 (6%)  183 (4%)  502 (11%)  34 (1%)  139 (3%)  134 (3%) | 243 (6%)  168 (4%)  431 (10%)  23 (1%)  101 (2%)  107 (3%) | 244 (6%)  179 (4%)  485 (12%)  38 (1%)  139 (3%)  130 (3%) |
| At least one respiratory allergy (m.d.=0) | 966 (33%) | 1,382 (33%) | 1,463 (33%) | 1,585 (33%) | 1,437 (34%) | 1,420 (34%) |
| BMI (m.d.=447)  Underweight  Normal weight  Overweight  Obese | m.d.=76  120 (4%)  1,680 (57%)  782 (27%)  296 (10%) | m.d.=98  190 (5%)  2,385 (58%)  1,069 (26%)  418 (10%) | m.d.=90  203 (5%)  2,488 (56%)  1,191 (27%)  467 (11%) | m.d.=81  200 (4%)  2,731 (57%)  1,260 (26%)  513 (11%) | m.d.=50  174 (4%)  2,397 (58%)  1,112 (27%)  471 (11%) | m.d.=52  166 (4%)  2,319 (56%)  1,157 (28%)  464 (11%) |

*Participants receiving a chronic treatment for at least one of the following diseases: asthma, diabetes, immunosuppression, heart, kidney, and pulmonary diseases.

m.d. = missing data.
