# Supplementary material for: Factors associated with influenza-like-illness: a crowdsourced cohort study from 2012/13 to 2017/18
Source: BMC Public Health. 2019 Jul 4;19:879. doi: 10.1186/s12889-019-7174-6 (PMC6610908; doi:10.1186/s12889-019-7174-6)
Supplement: Supplementary file 2 — Factors associated with at least one ILI episode (GNILI-) during six influenza epidemic periods, GrippeNet.fr. This table highlights the factors associated with an ILI episode using a more specific definition. (DOCX 25 kb) [file 12889_2019_7174_MOESM2_ESM.docx]

**Additional file 2.** Factors associated with at least one ILI episode (GN_ILI-)_ during six influenza epidemic periods, GrippeNet.fr

|  |  | **N*** | **Cases****  **n (%)** | **OR [CI 95%]**  **Univariate analyses** | ***p*-value** | **OR [CI 95%]**  **Multivariate analysis** | ***p*-value** |
| --- | --- | --- | --- | --- | --- | --- | --- |
| Season | 2012/13  2013/14  2014/15  2015/16  2016/17  2017/18 | 2,943  4,140  4,428  4,780  4,204  4,158 | 231 (8%)  111 (3%)  251 (6%)  203 (4%)  174 (4%)  198 (5%) | *Ref.*  0.32 [0.26;0.40]  0.70 [0.59;0.83]  0.51 [0.43;0.62]  0.50 [0.41;0.61]  0.58 [0.48;0.70] | <10^-4^ | *Ref.*  0.30 [0.24;0.38]  0.69 [0.58;0.83]  0.51 [0.42;0.62]  0.53 [0.43;0.65]  0.61 [0.50;0.74] | <10^-4^ |
| **Sociodemographic characteristics** | | | | | | | |
| Gender | Male  Female | 9,742  14,911 | 388 (4%)  780 (5%) | *Ref.*  1.33 [1.16;1.52] | <10^-4^ | *Ref.*  1.34 [1.17;1.53] | <10^-4^ |
| Age | [45-65)  [0-5)  [5-15)  [15-45)  [65-75)  ≥75 | 9,940  306  1,133  5,948  6,003  1,300 | 467 (5%)  20 (7%)  126 (11%)  330 (6%)  191 (3%)  33 (3%) | *Ref.*  1.43 [0.89;2.29]  2.50 [1.99;3.13]  1.19 [1.02;1.39]  0.67 [0.56;0.79]  0.53 [0.37;0.76] | <10^-4^ | *Ref.*  1.87 [1.14;3.07]  2.73 [2.14;3.49]  1.16 [0.98;1.36]  0.74 [0.61;0.89]  0.61 [0.42;0.90] | <10^-4^ |
| Household composition | Living alone  Living with ≥1 child  Living with adults only | 3,917  7,712  12,953 | 152 (4%)  501 (6%)  512 (4%) | *Ref.*  1.72 [1.42;2.08]  1.02 [0.85;1.23] | <10^-4^ | *Ref.*  1.36 [1.11;1.68]  1.08 [0.89;1.31] | <10^-2^ |
| Occupation | Working  Student  Unemployed  Retired  Stay at home/  Sick leave | 11,587  2,162  575  8,932  993 | 582 (5%)  191 (9%)  39 (7%)  288 (3%)  46 (5%) | *Ref.*  1.82 [1.51;2.20]  1.37 [0.96;1.95]  0.63 [0.54;0.73]  0.92 [0.67;1.27] | <10^-4^ | NS | NS |
| Place of residency | Rural  Urban | 4,840  19,813 | 231 (5%)  937 (5%) | *Ref.*  0.98 [0.84;1.15] | 0.84 | - | - |
| **Lifestyle** | | | | | | | |
| Use of public transport | No  Yes | 20,843  3,810 | 954 (5%)  214 (6%) | *Ref.*  1.24 [1.05;1.45] | <10^-2^ | NS | NS |
| Pets at home | None  At least one | 13,492  11,118 | 577 (4%)  588 (5%) | *Ref.*  1.23 [1.08;1.39] | <10^-2^ | *Ref.*  1.16 [1.02;1.32] | <10^-2^ |
| Contacts with patients | No  Yes | 22,163  2,490 | 1,024 (5%)  144 (6%) | *Ref.*  1.28 [1.06;1.54] | <10^-2^ | NS | NS |
| Contacts with elderly | No  Yes | 22,153  2,496 | 1,038 (5%)  130 (5%) | *Ref.*  1.14 [0.94;1.37] | 0.19 | *Ref.*  1.27 [1.05;1.55] | 0.03 |
| Contacts with a group of people | No  Yes | 16,795  7,858 | 746 (4%)  422 (5%) | *Ref.*  1.20 [1.06;1.36] | <10^-2^ | NS | NS |
| Contacts with children | No  Yes | 18,722  5,931 | 769 (4%)  399 (7%) | *Ref.*  1.65 [1.45;1.88] | <10^-4^ | NS | NS |
| **Health characteristics** | | | | | | | |
| Influenza vaccination (current season) | No  Yes | 16,083  8,554 | 835 (5%)  333 (4%) | *Ref.*  0.74 [0.65;0.85] | <10^-4^ | NS | NS |
| Influenza vaccination  (last season) | No  Yes | 15,190  9,305 | 792 (5%)  364 (4%) | *Ref.*  0.74 [0.65;0.85] | <10^-4^ | NS | NS |
| Smoking status | Non smoker  Smoker | 21,995  2,630 | 1,029 (5%)  138 (5%) | *Ref.*  1.13 [0.93;1.38] | 0.21 | - | - |
| Chronic treatment for at least one comorbidity*** | No comorbidities  At least one comorbidity  Asthma  Diabetes  Heart diseases  Kidney diseases  Immunosuppression  Pulmonary diseases | 19,425  5,228  1,449  941  2,473  152  655  635 | 899 (5%)  269 (5%)  110 (8%)  35 (4%)  98 (4%)  3 (2%)  39 (6%)  40 (6%) | *Ref.*  1.12 [0.96;1.29]  1.70 [1.37;2.11]  0.77 [0.54;1.11]  0.83 [0.66;1.03]  0.45 [0.16;1.28]  1.25 [0.89;1.75]  1.34 [0.95;1.88] | 0.15  <10^-4^  0.17  0.09  0.13  0.19  0.10 | *Ref.*  1.34 [1.14;1.57] | <10^-3^ |
| Respiratory allergy | None  At least one | 16,400  8,253 | 716 (4%)  452 (5%) | *Ref.*  1.25 [1.10;1.42] | <10^-3^ | *Ref.*  1.23 [1.08;1.41] | <10^-3^ |
| BMI | Normal weight [18.5;25[  Underweight <18.5  Overweight [25;30[  Obese ≥30 | 14,000  1,053  6,571  2,629 | 671 (5%)  57 (5%)  290 (4%)  129 (5%) | *Ref.*  1.12 [0.83;1.50]  0.92 [0.79;1.07]  1.03 [0.84;1.25] | 0.54 | - | - |

OR=Odd Ratio; CI95%=95% confidence interval; NS=not significant; - = variable not tested in the multivariate analysis as the p-value was higher than 0.2 in univariate analysis

*N=global number of individuals for the six GrippeNet.fr seasons (from 2012/13 to 2017/18), based on the 24,653 person-seasons. An individual may have participated up to six times (one time for each followed year).

**The ILI case definition used here is the GN_ILI-_ definition.

***Participants receiving a chronic treatment for at least one of the following diseases: asthma, diabetes, immunosuppression, heart, kidney, and pulmonary diseases. Inclusion of the gathered variable “At least one comorbidity” only in the analyses.
